# Mental health services in rural India: a big challenge still to be met

**DOI:** 10.1192/bji.2024.25

**Published:** 2024-11

**Authors:** Rahul Mathur, Nishtha Chawla, Rakesh K Chadda

**Affiliations:** 1 Department of Psychiatry, All India Institute of Medical Sciences, New Delhi, India, Email: drrakeshchadda@gmail.com; 2 JPNATC, All India Institute of Medical Sciences, New Delhi, India

**Keywords:** Mental health, rural, mental disorders, primary care, tele mental health

## Abstract

There has been a decline in the rural population of India from nearly 82% to about 65% over the past six decades. The National Mental Health Survey of India (2015–2016) reported a lower prevalence of mental disorders in rural areas compared with urban ones. Mental health services in the country are skewed towards the urban areas, and more families are pushed below the poverty line while getting treatment for a member with mental illness. India has expanded its District Mental Health Programme over the past two decades, and it now covers nearly all the districts in the country. Despite that, significant numbers of people with mental disorders, ranging from 70–90%, do not receive adequate treatment. This paper discusses the rural–urban divide in the mental health services, examining the problem and need, and the initiatives taken by the government of India in this direction.

## The rural–urban divide

India, located in the southern part of the Asian continent, is a vast nation with an area of 3.287 million km^2^ and a population of about 1.42 billion. The population of India shows wide variation and diversity across different regions. A substantial portion of the population resides in rural areas, although there has been an increasing trend towards urbanisation since the country's independence in 1947. The rural population is split between villages and small towns, where most of the people work in agriculture or other rural occupations. According to the World Bank collection of development indicators, the rural population in India accounted for 64.13% of the total population in 2022; this is similar to the share of rural population in other South Asian countries (Bhutan 56.31%, Bangladesh 60.29%, Pakistan 62.27%, Nepal 78.55% and Sri Lanka 80.97%). The rural population of India has increased 2.5-fold in the 60 years from 1961 to 2021, while the proportion of the rural population declined from 81.97 to 68.72% (Table 1).

**Table 1:** Rural population of India (World Bank data)

Year	Rural population (in millions)	Percentage of total
1961	374.1	81.97
1971	456.1	80.01
1981	545.9	76.58
1991	659.8	74.22
2001	777.7	72.08
2011	864.3	68.72
2021	909.4	64.61

The rural–urban mental health divide presents unique challenges. In rural areas, shifting family dynamics and urban migration disrupt traditional support systems for individuals with mental health concerns. A lack of mental health infrastructure exacerbates the issue, with limited availability of professionals and resources. In addition, substance use is widespread, further affecting mental health. This compounds the need for effective rural mental health services, especially against the background of the changing family structures and urban migration. Previous literature has warned that numbers of mental health patients in rural areas will rise significantly over the next decade, owing to the growing population, shifting values, changing lifestyles, crop failures, natural disasters (drought and flood), economic crises, unemployment, lack of social support and rising insecurity.^1^ This paper discusses the nature and extent of mental health problems in rural versus urban populations in India, as well as the rural–urban divide in the mental health services and the initiatives taken by the Government of India in this direction.

## Burden of mental illness in India

**Fig. 1:**
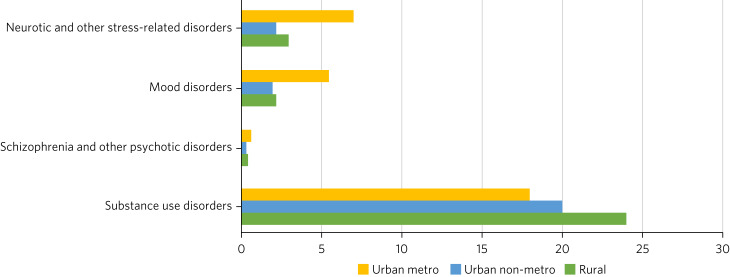
Prevalence (%) of mental disorders in rural versus urban population (source: NMHS of India 2015–2016).

The National Mental Health Survey (NMHS) of India (2015–2016) found variations in the prevalence of mental disorders across urban and rural areas.^2^ Mental disorders were found to be twice as prevalent in urban areas compared with rural areas (13.5% *v.* 6.9%). However, the burden of substance use disorders, predominantly involving alcohol and tobacco, was higher in the rural population. Other mental disorders (Fig. 1), including schizophrenia, mood disorders and neurotic/stress-related disorders, were two to three times more prevalent in urban areas.^3^ Other national level surveys have also found differences in the distribution of various psychiatric and/or substance-related illnesses. For example, according to the Global Adult Tobacco Survey 2, National Family Health Survey 5 (2019–2021) and National Non-communicable diseases Monitoring Survey (NNMS; 2018), the prevalence of tobacco use in rural areas (approximately 35–42%) was much higher than that in urban areas (approximately 20–25%). The NNMS also found the prevalence of alcohol use to be slightly higher in rural than urban areas (17% *v.* 14%).^3^ These variations in prevalence have been hypothesised to be caused by either natural variation or variations in cultural understanding and reporting of symptoms suggestive of mental illness.

The treatment gap, as highlighted in the NMHS 2015–2016, was found to range from 70–92%, with the greatest treatment gap (91.8%) for tobacco use disorder.^2^ These wide treatment gaps are critically influenced by lack of awareness and limited availability of mental health services, especially in rural areas, and poor affordability.

Thus, mental disorders are prevalent in urban areas, but rural areas also have significant numbers of people in need of care owing to limited availability of services. Various factors may contribute to the reported differences, including differences in stress levels, complexity of living situations, level of support systems, changing lifestyles, economic or agricultural challenges, and other issues that may affect mental health. Equitable mental health service coverage is vital to address these disparities. Ensuring equal access to mental health services in both urban and rural areas is crucial to meet the diverse needs of the population.

## Mental health resources in India

According to the World Mental Health Atlas (2017),^4^ India falls far short of recommended mental health standards. There are only 0.20 beds per 10 000 population, and 0.29 psychiatrists, 0.07 clinical psychologists, 0.8 psychiatric nurses and 0.06 social workers per 100 000 population, highlighting the inadequacy of mental health support. A rough estimate of the number of psychiatrists in India in the current year (2024) would be 0.7 per 100 000 population. The World Mental Health Atlas estimated that high-income countries have 120 times more psychiatrists than low-income countries, with a global median of 1.3 psychiatrists per 100 000 population.

With respect to the health infrastructure, numbers of government hospital beds in rural areas are less than half of those available in urban areas. Further, the bed/population ratio in rural areas is one-fifth of that in urban areas.^1^ Regarding mental health infrastructure, India has 952 hospital-based, 1217 community-based and 240 out-patient mental health facilities, and 139 mental health facilities for children and adolescents.^4^ In addition, there are 136 mental hospitals, 389 general hospital psychiatric units and 223 long-term care facilities, mostly in urban areas. However, despite progress, the country still lags behind the World Health Organization (WHO) recommended standard of one psychiatrist per 100 000 population. It is important to mention here that in the past 10 years, there has been a substantial increase in the training opportunities for psychiatrists as a part of an initiative taken by the local government. This should help to achieve the WHO recommended standard as a whole for the country, although the rural–urban divide in services may continue to be a big challenge.

## Beginning of rural mental health services in India

In the mid-1960s, rural mental health services were starting to be developed in India.^5^ In 1964, a weekly service was started at the Comprehensive Rural Hospital in Ballabhgarh, a small town near Delhi; this was followed by another community clinic in Mandar village near Ranchi, in eastern India, in 1967. In the 1970s, community psychiatry advanced, with medical colleges establishing clinics in collaboration with the Departments of Community Medicine. There were two key initiatives at Raipur Rani block in Haryana in North India and in Sakalwada village in Karnataka in South India, which acted as forerunners of the National Mental Health Programme of India in 1982, expanding mental health services in rural India, bridging accessibility gaps and providing training for healthcare professionals to address rural mental health challenges. Major roadblocks remained in the form of limited budgetary support, inadequate staff, lack of political commitment and lukewarm response among mental health professionals.^5^ However, a major achievement followed with the evolution of the district model of providing mental healthcare, leading to the launch of the District Mental Health Programme (DMHP) in 1996 (discussed in the next section).

## Current status of mental health services in rural India

According to data acquired from a national sample survey of India in July to December 2018, which also estimated catastrophic health expenditure and poverty impact due to mental illness, 22.5% of households in rural areas fell below the poverty line while a family member with mental illness underwent treatment, compared with 17% in urban areas (17%).^6^

Mental health services in India are limited, with progress primarily in some southern states such as Kerala, where psychiatric care is widely available. In most other places in the country, services in the community are offered through extension clinics at medical schools or under the DMHP. Private psychiatrists hold occasional clinics in some areas, addressing the accessibility gap. In addition, voluntary organisations and private psychiatrists organise community camps to provide on-site mental health services to rural communities, addressing their specific needs.^5^

In Karnataka, the DMHP has implemented several significant initiatives to strengthen mental healthcare services in India.^7^ These initiatives represent a comprehensive approach to addressing various aspects of mental health support. The Manochaitanya Programme, also known as ‘Super Tuesdays’, offers specialised out-patient clinical services at Taluk hospitals, enhancing local access to expert mental healthcare. The Maanasadhara Programme provides day care rehabilitation services for individuals with severe mental illnesses, aiding their recovery and daily functioning. ‘Manasakendras’ serve as halfway homes, offering structured support for individuals transitioning from psychiatric institutions to community living. The Assisted Home Care Services Programme reaches out to individuals who have dropped out of treatment owing to severe mental illnesses, with mental health professionals conducting home visits to ensure continuity of care and medication adherence.^8^ The Primary Care Psychiatry Programme focuses on training and supporting primary care doctors to identify and manage mental illnesses, ultimately strengthening early intervention and outcomes.^9^ These initiatives collectively aim to enhance mental healthcare services in India and improve outcomes for individuals with mental illnesses throughout the country.

Telemedicine and telepsychiatry initiatives have also emerged as valuable tools in extending mental health services to remote areas. As part of the initiative, Tele MANAS (Tele Mental Health Assistance and Networking Across States) was launched on World Mental Health Day in 2022, promising a robust tele mental health infrastructure and network across the country that would make mental health services readily available and accessible to all.^10^ By providing round-the-clock support and comprehensive interventions and extending services to vulnerable populations, the programme aims to address the mental health needs of the population and ultimately improve mental health outcomes throughout India. Through telemedicine platforms such as Tele MANAS, individuals in rural communities can access mental health consultations and support remotely, overcoming geographical barriers. Specific pilot projects have been undertaken by psychiatrists, focusing on innovative approaches to deliver mental health services in rural areas.

Another important initiative has been the Systematic Medical Appraisal, Referral and Treatment Mental Health Project in rural areas in the West Godavari district of Andhra Pradesh, India. This involved training primary healthcare physicians, ASHAs (accredited social health activists) and project staff in screening, diagnosing and treating depression, suicide risk and emotional problems using an electronic decision support system tool.^11^ Recently, the ESSENCE (enabling translation of science to service to enhance depression care) project in Madhya Pradesh, India, has been planned to address the issue of limited capacity by training large cadres of frontline workers in low- and middle-income countries through two consecutive randomised controlled trials.^12^ These projects serve as experimental models to explore and evaluate new methods of effectively reaching underserved populations.

Although progress has been made in expanding mental health services in rural India, challenges such as limited resources, infrastructure and awareness persist. However, these ongoing initiatives demonstrate a commitment to improving mental healthcare accessibility and addressing the unique needs of rural communities in India. A study from rural south India assessing the impact of community-based rehabilitation for mental illness on out-of-pocket expenditure in households with a person with severe mental illness found that the average annual cost per person decreased to Indian rupees (Rs) 492 (US$7) with community-based rehabilitation. The net annual savings for the system for 95 persons with severe mental illness covered in the study was Rs 383 755 (US $5482).^13^

## Roadblocks

The roadblocks to providing mental health services in rural India include limited infrastructure, lack of adequate facilities, limited funding for mental health, workforce and inequitable distribution of resources. Stigma, discrimination, traditional and religious beliefs, and sociocultural barriers form a background for reluctance in seeking help. Low mental health literacy and lack of awareness also contribute to underutilisation of existing services. Geographical barriers, especially in hilly and tribal areas, together with poor transportation infrastructure affect access to services. Last, questionable political will, unclear plans and/or policies regarding mental health, poor training of the general health workforce, inadequate integration of mental health in primary healthcare, lack of public health skills among mental health leaders, and overburdened primary healthcare also contribute to fragmented care.^1^

## Conclusion and way forward

To bridge the rural–urban mental health divide, a holistic approach is needed. Initiatives have been taken at various levels in India in this regard. Steps towards improving rural mental health services include enhancing mental health infrastructure, boosting numbers of rural mental health professionals and integrating services into primary healthcare. In addition, awareness-raising and provision of specific mental health interventions in rural areas are vital for equitable access to mental healthcare. ASHA workers are an important resource in rural areas and are involved in various national programmes. Capacity-building and training of ASHA workers to identify mental illnesses and participate in rehabilitation may be a productive step in the right direction. Other resources include traditional healers who are well trusted in rural communities. These two strategies can contribute to early identification, improved treatment-seeking and subsequent rehabilitation at a rural level.^1^

The facilitators to providing mental health services in rural India include telepsychiatry, which offers a cost-effective way to expand services in remote areas. Community engagement, involving local leaders, health workers and traditional healers, can raise awareness and facilitate service delivery. Tailoring of interventions to align with local cultural norms and beliefs enhances their acceptability and effectiveness. Integration of mental health services into primary care settings improves access and reduces stigma. Public health education programmes and awareness campaigns help to dispel myths and promote help-seeking behaviours. Innovative models of care delivery, such as the Raipur Rani project and the Sakalwara model, demonstrate the feasibility of providing services in rural areas. Integration of mental health with non-communicable diseases is a welcome step by the WHO, as it is likely to reduce stigma and subsequently the treatment gap in underprivileged and low-resource areas.
